# Experimental Study and Mechanism Analysis of the Flow Boiling and Heat Transfer Characteristics in Microchannels with Different Surface Wettability

**DOI:** 10.3390/mi12080881

**Published:** 2021-07-27

**Authors:** Shengnan Zhou, Bifen Shu, Zukang Yu, Yan Huang, Yuqi Zhang

**Affiliations:** Guangdong Provincial Key Laboratory of Photovoltaic Technology, School of Physics, Sun Yat-sen University, Guangzhou 510006, China; zhoushn@mail2.sysu.edu.cn (S.Z.); yuzk3@mail2.sysu.edu.cn (Z.Y.); huangy235@mail2.sysu.edu.cn (Y.H.); zhangyq69@mail2.sysu.edu.cn (Y.Z.)

**Keywords:** flow boiling, surface wettability, heat transfer mechanism, flow patterns, dryout phenomenon

## Abstract

In this paper experiments have been conducted to investigate the flow boiling and heat transfer characteristics in microchannels with three different surface wettability. Three types of microchannels with a super-hydrophilic surface (θ ≈ 0°), a hydrophilic surface (θ = 43°) and an untreated surface (θ = 70°) were prepared. The results show that the average heat transfer coefficient of a super-hydrophilic surface microchannel is significantly higher than that of an untreated surface microchannel, especially when the mass flux is high. The visualization of the flow patterns states that the number of bubble nucleation generated in the super-hydrophilic microchannel at the beginning of the flow boiling is significantly more than that in the untreated microchannel. Through detailed analysis of the experimental data, flow patterns and microchannel surface SEM images, it can be inferred that the super-hydrophilic surface microchannel has more active nucleation cavities, a high nucleation rate and a large nucleation number, a small bubble departure diameter and a fast departure frequency, thereby promoting the flow and heat transfer in the microchannel. In addition, through the force analysis of the vapor-liquid interface, the mechanism that the super-hydrophilic microchannel without dryout under high heat flux conditions is clarified.

## 1. Introduction

Two-phase flow boiling microchannels have appeared as one of the most efficient solutions for modern high heat flux cooling applications [1,2,3], including computer electronics, x-ray medical devices, satellite and spacecraft avionics and so on. Despite this broad range of applications, two phase microchannels display many fundamental issues in flow boiling, which are heat transfer characteristics, dominant heat transfer mechanism, flow patterns, critical heat flux, pressure drop and flow instabilities. Improving the heat transfer coefficient and increasing the critical heat flux is a primary motivation. Recent studies show that surface modification technology [4] is an effective way to accomplish this.

Yang et al. [5] prepared super-hydrophilic silicon nanowires based on the inner wall of the microchannel, and conducted flow boiling experiments using deionized water as the experimental working medium. Studies had shown that in the annular flow region, in addition to nucleate boiling and thin film evaporation, there was also liquid film replacement. In addition, it was revealed that due to the super-hydrophilic surface of the microchannels, there were sediments on the surface, firstly it was easy to promote capillary flow and liquid renewal, and powerfully enhanced thin film evaporation and liquid film replacement. Secondly, the separation of vapor-liquid two-phase flow interface could reduce the signs of droplet entrainment and energy consumption, and optimize heat transfer performance [6]. Liu et al. [7] investigated silicon-based microchannel surfaces with contact angles of 0°, 36° and 103° produced by plasma etching and growth of nanowires, and experimentally studied flow boiling under the conditions of heat fluxes in 230–354.9 kW/m^2^ and mass fluxes in 50–583 kg/(m^2^s). It was observed that the liquid on the hydrophobic surface was seriously overheated. Once the bubbles were nucleated, they would expand violently, which was not conducive to the nucleation of bubble. However, the hydrophilic surface had bubble nucleation and growth detachment, while the super-hydrophilic surface showed excellent bubble nucleation sites.

In the early stage of boiling, bubble nucleation is certainly important, but in the middle and late stages of boiling, the phenomenon of local dryout easily emerged with increasing heat flux or vapor quality, which made the wall surface heat up rapidly and burnt the equipment. How to solve the problem of frequent dryout and improve the heat exchange performance of the microchannels in flow boiling was particularly critical [8,9,10,11]. Zhou et al. [12] examined the impact of surface wettability with contact angles of 65° and 0° on deionized water flow boiling in microchannels. Flow visualization indicated local dryout occurring on the untreated surface at high heat flux and low mass flux, attached to deterioration in heat transfer performance. Conversely, this tendency was not observed on a super-hydrophilic surface, which was consistent with recent research conclusions [13,14]. However, a mechanism for alleviating the dryout phenomenon needed further study.

Research has not reached extensive and universal conclusions about the dominant heat transfer mechanisms of normal microchannels. Besides, the mechanism of super-hydrophilic surfaces was less studied. For instance, several researchers [15,16,17] have reported that nucleate boiling was the prevailing heat transfer mechanism, but various researchers [18,19,20,21] concluded that convective boiling was the foremost heat transfer mechanism. Some researchers [22,23] described that the nucleate boiling mechanism dominated at low vapor quality, while convective boiling mechanism guided the process at high vapor quality. These studies [24,25,26,27] used conventional criteria to deduce the leading heat transfer mechanism, which was heat transfer coefficient dependent on heat flux in nucleate boiling while it relied on vapor quality and mass flux in convective boiling. In addition to the influence of heat flux, vapor quality, pressure and mass flux, some researchers also studied the effects of channel shape and flow patterns [28]. Moreover, based on the fact that slug flow and annular flow accounted for the main flow patterns, some researchers inferred that the evaporation process of thin liquid film was an important part of phase change heat transfer in microchannels [29,30].

The above literature review demonstrates that the influence of surface wettability on flow boiling is complicated, and the heat transfer mechanism of microchannel is also controversial. More research is still required to understand the flow boiling enhanced heat transfer characteristics and mechanisms in super-hydrophilic microchannel compared with untreated microchannel, which is a main motivation for the present work, which emphasizes the effect of heat flux, inlet vapor quality and mass flux on the average heat transfer coefficients of aluminum-based microchannels of different surface wettability with R134-a as working fluid. Flow boiling data were obtained for heat fluxes of 0–46 kW/m^2^, mass fluxes of 740–1265 kg/(m^2^s), inlet vapor qualities of 0–0.25. Moreover, the advantages and heat transfer mechanism of super-hydrophilic microchannel are further explored by utilizing flow visualization, in particular the reason of the bubble formation, avoiding dryout and the flow pattern transition.

## 2. Materials and Methods

### 2.1. Flow Loop

**Figure 1:**
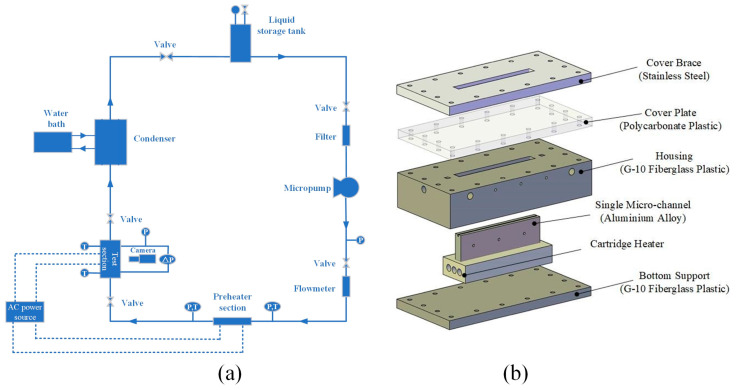
(**a**) Schematic diagram of the system device, (**b**) CAD images of the test section.

A vapor-liquid two-phase flow experimental system is constructed. A schematic diagram of the system device is shown in Figure 1a, which is referred to prior research [31] with further improvements. The system is composed of a circulating loop, using R-134a refrigerant as the experimental working fluid. The working fluid first flows from the liquid storage tank to the inside of the experimental loop, and then flows through the filter, gear pump and volume flow meter. The gear pump is the energy and power source of the entire experimental device and provides the driving force for the flow of the working fluid. The flow meter is used to read the volume flow of the working fluid, which is convenient for adjusting experimental parameters such as mass flux. In addition, the working fluid adjusts its inlet vapor quality through the preheating of the preheating section, and then the working fluid flows through the microchannel test section for heat exchange, and finally condenses back to the liquid storage tank through the condensation system to form a complete circulation loop system. Among them, the preheating section and the testing section are heated by electric heating to ensure the uniformity of heat flux. The liquid storage tank is placed in an environment with a room temperature of 20 °C, and the pressure of the liquid storage tank is adjusted to ensure that the working fluid flowing out of the liquid storage tank is in a saturated liquid state.

In order to efficiently circulate the reflux, the condenser system adopts a totally enclosed water-cooling device, and the temperature is set to 20 °C, which is consistent with room temperature. In addition, for the purpose of reducing heat loss, the entire experimental device is wrapped with asbestos. In the circulating flow process, the experimental parameters such as pressure and temperature are read and recorded by the data acquisition instrument. At the same time, the working conditions in different ranges are adjusted by adjusting the working fluid flow and heating power of the preheating section and the test section. In order to more conveniently and meticulously capture the situation and dynamic transition process of the flow patterns inside the microchannel, a high-speed camera is used to build a visualization system at the test section.

The experimental test section is shown in Figure 1b. The microchannel is based on an aluminum base, and the length achieved through machining is 78 mm, the width is 1.6 mm, and the height is 0.6 mm. The size parameters of the microchannel are shown in Table 1.

Thermocouples are installed at the entrance and exit of the microchannel to facilitate the measurement of the temperature of the entrance and exit chamber. A K-type thermocouple could monitor the local wall temperature fluctuations of the microchannel. In addition, the pressure sensor tracks the working fluid based on the initial pressure of the liquid storage tank and the inlet pressure of the test section, while the differential pressure transmitter collects the data of inlet and outlet pressure difference of the test section.

### 2.2. Preparation and Characterization of Different Surface Wettability Microchannels

**Figure 2:**
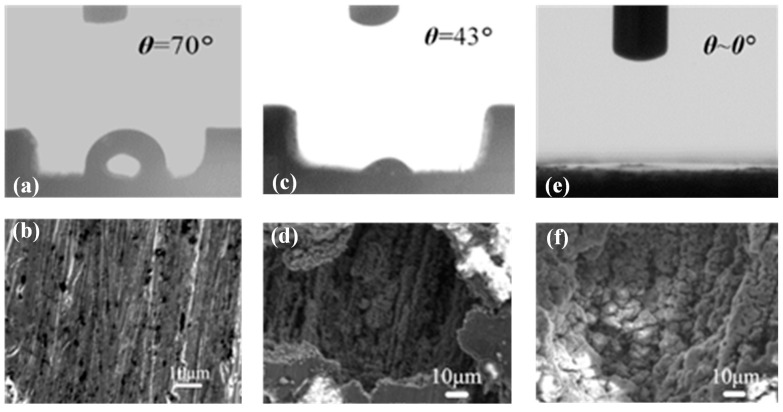
Contact angles and SEM images of different surfaces for (**a**,**b**) Untreated surface microchannel, (**c**,**d**) Hydrophilic microchannel, (**e**,**f**) Super-hydrophilic microchannel.

Three kinds of 78 mm × 1.6 mm × 0.6 mm aluminum-based microchannels were successfully fabricated by electro discharge machining or micro-milling [32], grinding, chemical etching and ultrasonic cleaning, with untreated surface (θ = 70°), complex hydrophilic surface (θ = 43°) and super-hydrophilic surface (θ ≈ 0°). Their surface contact angles are shown in Figure 2a,c,e. Moreover, the surface morphologies of different wettability microchannels were obtained by SEM, as shown in Figure 2b,d,f. It can be seen from the Figure 2b,f that the untreated surface is smooth and flat, while the super-hydrophilic surface is rough and has many active cavities.

### 2.3. Data Processing

In this paper, R-134a refrigerant is used as the experimental working fluid, and the two-phase flow boiling experiment is carried out at a room temperature of 20 °C. The inlet and outlet temperature of the working fluid and the wall temperature of the test section are measured by pt100 and K-type thermocouples. After calibration, the measurement errors of the two thermocouples are within 0.5 °C and 0.3 °C, respectively. The working fluid pressure and differential pressure are respectively measured by a pressure sensor and a differential pressure transmitter. After calibration, the measurement error of the pressure sensor and the differential pressure transmitter is less than 0.5%. The experimental conditions are shown in Table 2, and the experimental errors and uncertainties are shown in Table 3.

In the experiment, the refrigerant flows out of the liquid storage tank which is saturated. Since the change of heat transfer and pressure drop between the initial pressure measurement position of the liquid storage tank and the pump outlet is negligible, it could be assumed that the refrigerant in this section is in saturated liquid state. Along the pipeline from the initial pressure measurement to the microchannel inlet, the pump speed and room temperature are constant, which indicates that the mass flow in the microchannel is a fixed value. The inlet vapor quality would change when the preheating power is adjusted. According to the conservation of energy, the inlet vapor quality is calculated as:

(1) $$x_{in}=\frac{h_{f,res}+P/\dot{m}-h_{f,in}}{h_{fg,in}}$$

where $h_{f,\mathrm{res}}$ is the saturated liquid enthalpy based on the pressure of the liquid storage tank, P is the heating power at the preheating section, $\dot{m}$ is the mass flux, $h_{\mathrm{fg},\mathrm{in}}$ is the latent heat of vaporization of the working fluid at the inlet.

In the experimental test section, the working fluid is heated and its vapor quality becomes larger, and the heat absorbs by it is moved away from latent heat of vaporization of the phase change. Since the surface roughness of the microchannel is uniform, it is assumed that the internal pressure drop in the channel is also uniform, that is the pressure at any position could be determined by the inlet pressure. According to the principle of thermal balance, the local vapor quality of the microchannel is:

(2) $$x_{z}=x_{in}+\frac{Q_{test}}{\dot{m}h_{fg,z}}\times \frac{z}{L}$$

where $Q_{\mathrm{test}}$ is heating power in test section, $h_{\mathrm{fg},z}$ is the latent heat of vaporization of the working fluid at any position inside the channel. Local saturated pressure $P_{\mathrm{sat},z}$ is calculated as following formulation:

(3) $$P_{sat,z}=P_{sat,in}-\Delta P\frac{z}{L}$$

The two-phase local heat transfer coefficient is calculated as follows:

(4) $$h_{z}=\frac{q^{\prime \prime }}{T_{W,z}-T_{sat,z}}$$

where $T_{W,z}$ is the local wall temperature, $q^{\prime \prime }$ is the effective heat flux and $T_{\mathrm{sat},z}$ is the saturated temperature bases on the local saturated pressure.

The average heat transfer coefficient is as follows:

(5) $$h=\frac{1}{L}\int_{0}^{L}h(z)dl$$

## 3. Results and Discussion

### 3.1. Influence of Surface Wettability on Average Heat Transfer Coefficients

**Figure 3:**
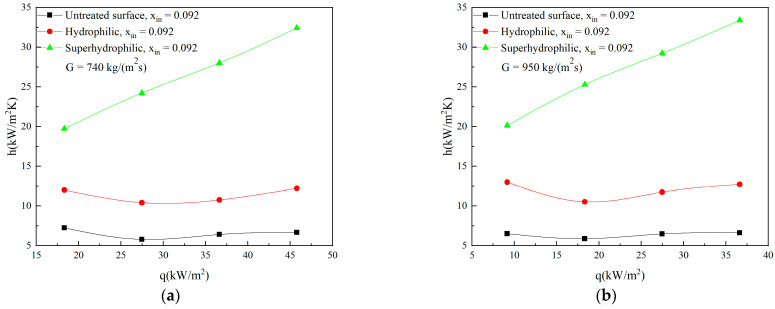

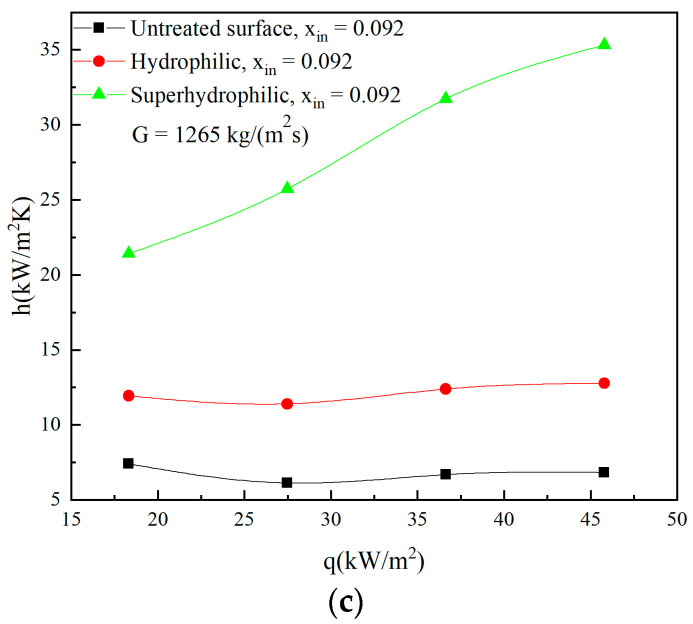
The effect of surface wettability on average heat transfer coefficients with the increase of heat fluxes for (**a**) G = 740 kg/(m^2^s), (**b**) G = 950 kg/(m^2^s), (**c**) G = 1265 kg/(m^2^s).

The heat transfer performance of the microchannels heat exchanger is mainly characterized by the average heat transfer coefficients. Figure 3a–c show the average heat transfer coefficients versus heat fluxes in microchannels with different wettability under different inlet vapor qualities and mass fluxes conditions. First of all, it can be seen that the heat transfer performance of the super-hydrophilic surface microchannel is much better than that of the untreated surface microchannel, and the advantage is more significant at higher mass flux. At a lower mass flux of 740 kg/(m^2^s), the average heat transfer coefficients of hydrophilic and super-hydrophilic microchannels are about 65% and 300% higher than those of untreated surface microchannel, respectively. However, the average heat transfer coefficients of hydrophilic and super-hydrophilic microchannels increase by 85% and 325% compared with those of untreated surface microchannel at higher mass flux of 1265 kg/(m^2^s), respectively. It shows that the heat fluxes have more significant effect on the heat transfer characteristics of super-hydrophilic microchannel in the high mass flux region.

Secondly, the heat transfer coefficients of super-hydrophilic surface microchannel increase significantly as the heat fluxes increase, while the heat transfer coefficients of untreated and hydrophilic microchannels change little. This reveals that the super-hydrophilic surface microchannel has significant effects on improving not only the heat transfer coefficients, but also the heat fluxes of the micro heat exchanger.

### 3.2. Advantages of Super-Hydrophilic Microchannel

Due to the importance of high heat flux demand for micro heat exchanger, this section focuses on the influence of different heat fluxes on the heat transfer coefficients, especially the critical heat flux when the phenomenon of dryout occurs.

**Figure 4:**
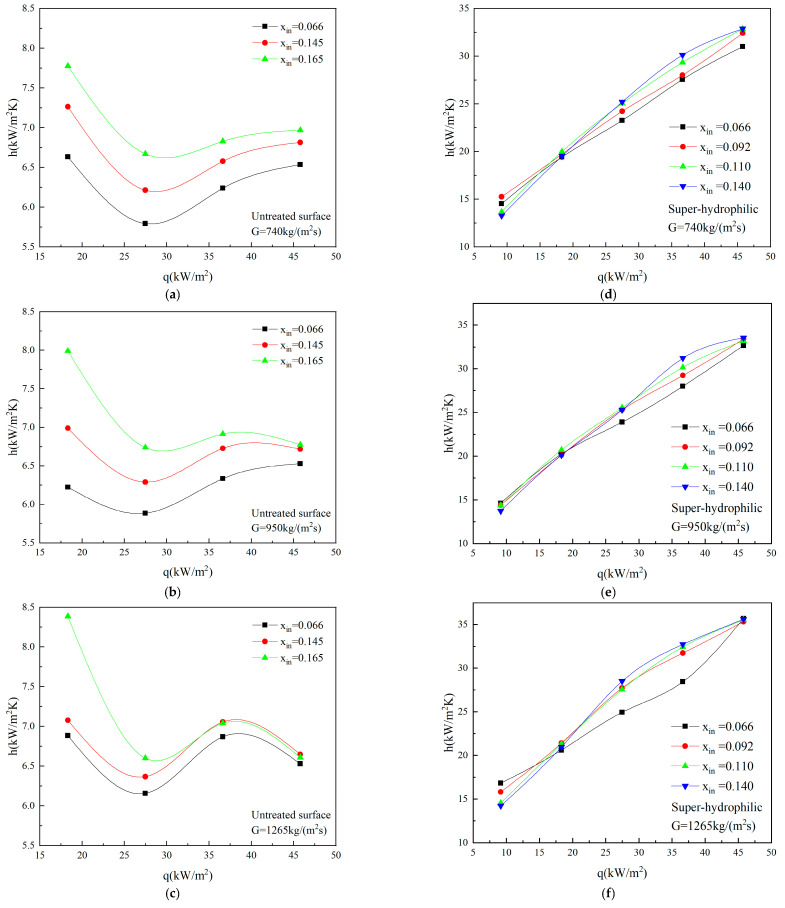
The variation trend of average heat transfer coefficients with the increase of heat fluxes under different inlet vapor qualities for (**a**–**c**), untreated surface microchannel and (**d**–**f**), super-hydrophilic microchannel.

**Figure 5:**
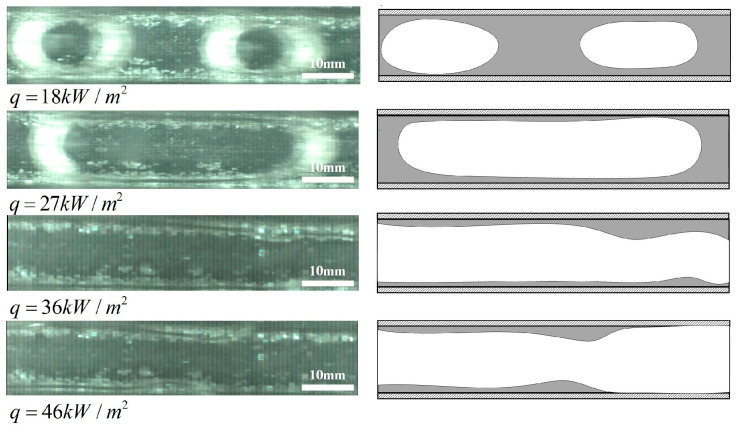
Two phase flow patterns of untreated surface microchannel under different heat fluxes for G = 1265 kg/(m^2^s), x_in_ = 0.066.

For untreated surface microchannel, the average heat transfer coefficients versus heat fluxes with different inlet vapor qualities at mass fluxes of 740 kg/(m^2^s), 950 kg/(m^2^s) and 1265 kg/(m^2^s) are shown in Figure 4a–c, respectively. It can be seen that with the increase of heat fluxes, the average heat transfer coefficients first decrease, then increases, and decreases again. By observing the flow patterns shown in Figure 5, bubble nucleation, growth and departure occur when q ≤ 27 kW/m^2^, which indicates strong heat transfer characteristics. With the increase of heat fluxes, the growth and expansion of bubble are restricted due to the channel size effect, and the flow patterns mainly change from bubble flow to slug flow/plug flow. In the slug flow region, with the increase of heat fluxes, density of nucleation and departure frequency of bubble are limited by the liquid film, and the departure diameter increases, which lead to the sharp decrease of heat transfer coefficients. Thus, at low heat fluxes, nucleate boiling dominates the heat exchanger. When 27 kW/m^2^ < q ≤ 36 kW/m^2^, the heat transfer coefficients increase with the increase of heat fluxes. Under this work condition, the flow patterns show mainly annular flow, and the liquid film is gradually formed. However, as the heat fluxes continue to increase in the region of q > 36 kW/m^2^, the liquid film becomes very thin, and even appears the phenomenon of lack of liquid, resulting in local dryout patches. It is found that in the region of high heat fluxes and high vapor qualities, the larger the heat fluxes are, the larger the dryout areas are, the more obvious the phenomenon is and the worse the heat transfer is. Therefore, at high heat fluxes, convective boiling is dominant. This is consistent with the conclusion of Mudawar et al. [27] and Yang et al. [31].

**Figure 6:**
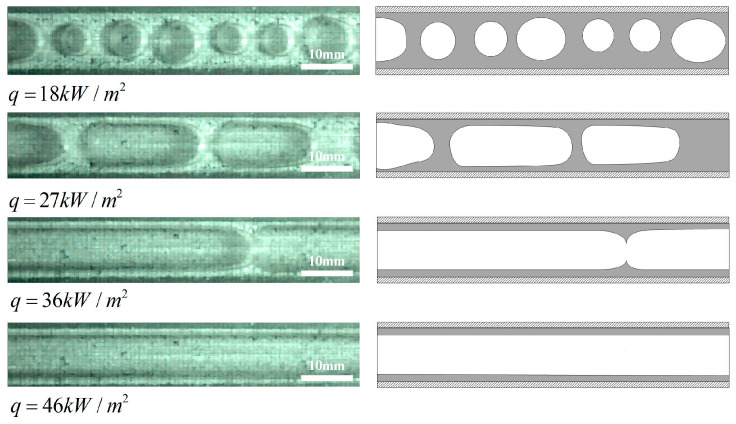
Two phase flow patterns of super-hydrophilic microchannel under different heat fluxes for G = 1265 kg/(m^2^s), x_in_ = 0.066.

For a super-hydrophilic microchannel, the relationship between the average heat transfer coefficients and the heat fluxes with the same mass flux and different inlet vapor qualities is shown in Figure 4d–f. It can be noticed that the average heat transfer coefficients of super-hydrophilic surface microchannel increase with the increase of heat fluxes, basically showing a linear positive correlation, which is obviously different from those of the untreated surface microchannel. When q ≤ 27 kW/m^2^, through the analysis of the two-phase flow patterns shown in Figure 6, the flow patterns show mainly bubbly flow and slug flow/plug flow. Besides, bubble nucleation density and departure frequency increase, and departure diameter decreases, which lead to the improvement of the heat transfer performance. Under this work condition, the heat exchange is dominated by nucleate boiling. When q > 27 kW/m^2^, it is found that there is no local dryout phenomenon on the super-hydrophilic surface. In this region, the flow patterns display mainly annular flow. It is speculated that the super-hydrophilic surface has better re-wettability, there is enough liquid for replenishment, the liquid film distribution is uniform, and the heat transfer is enhanced, so at high heat flux, the heat exchange mechanism is speculated to be thin liquid film evaporation. In this experiment, it is further confirmed that in the flow boiling in microchannels at high heat flux, the super-hydrophilic surface could delay or even avoid the occurrence of local dryout, thereby increases the critical heat flux, which is consistent with the results of Zhou et al. [12]. This advantage of super-hydrophilic surface is expected to play a vital role in the practical application of high heat flux density microchannel two-phase heat dissipation system.

In addition, the influence of different inlet vapor qualities on the heat transfer coefficients is discussed. According to the analysis of Figure 4–c, it is found that in the studied range of experimental working conditions, the average heat transfer coefficients increase with the increase of inlet vapor qualities for untreated surface microchannel. In the high heat flux region of q > 27 kW/m^2^, the inlet vapor qualities have a relatively small influence on the heat transfer coefficients, while at low heat flux region of q < 27 kW/m^2^, the inlet vapor qualities have a greater influence on the heat transfer coefficients. It is speculated that the increase of vapor qualities promoted the nucleation rate of bubbles and increased the number of bubbles. With the increase of the amount of bubble, the first the ONB is advanced, and the single-phase flow enters the two-phase flow area more quickly. The second is enhancement the movement of the liquid film, the third is to speed up the flow rate between vapor and liquid to enhance heat transfer.

However, it is found from Figure 4d–f that for a super-hydrophilic microchannel, the inlet vapor qualities have a greater influence on the heat transfer coefficients at high heat fluxes than that at low heat fluxes. The analysis show that this is due to the excellent rewetting characteristics of super-hydrophilic surface, which is more prominent under the influence of inlet vapor qualities. Secondly, it is acknowledged that at low heat fluxes of q < 18 kW/m^2^, the smaller the inlet vapor qualities are, the larger the heat transfer coefficients are. When the heat fluxes of q > 18 kW/m^2^, the greater the inlet vapor qualities are, the greater the heat transfer coefficients are. The reason is that only a small degree of inlet vapor quality could promote nucleate boiling and enhance the heat transfer at lower heat fluxes. The larger the vapor qualities are, the larger the departure diameter is and the smaller the heat transfer coefficients are. At high heat fluxes, the increase of vapor qualities leads to the increase of bubble number and promote the movement of liquid film. Moreover, the surface tension of vapor-liquid interface plays a leading role.

### 3.3. Influence of Surface Wettability on Flow Patterns

**Figure 7:**
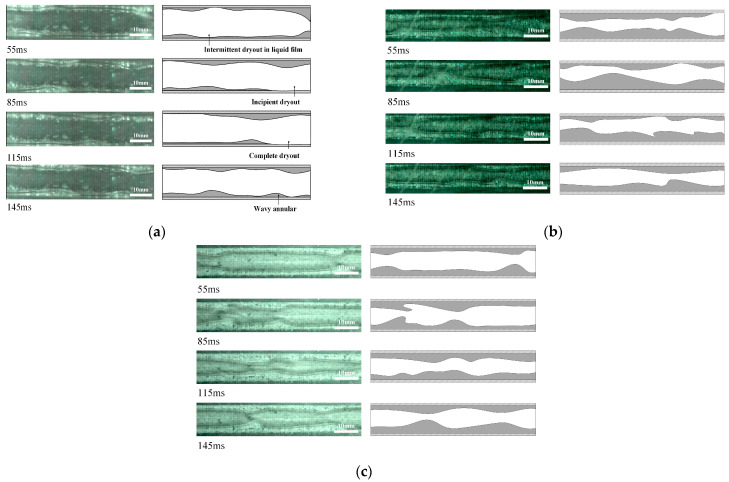
Comparison of flow patterns in microchannels with different wettability under G = 950 kg/(m^2^s), x_in_ = 0.066, q = 46 kW/m^2^ for (**a**) Untreated surface microchannel, (**b**) Hydrophilic microchannel, (**c**) Super-hydrophilic microchannel.

The two-phase flow patterns could further explain the heat transfer mechanism from the aspects of liquid flow state, bubble dynamics and phase interface distribution. In order to further reveal the influence of surface wettability on the two-phase flow patterns, especially in the stage of dryout the flow patterns under the working conditions of G = 950 kg/(m^2^s), x_in_ = 0.066, q = 46 kW/m^2^ are further observed and analyzed. It is found from Figure 7a that intermittent dryout occurs in the annular flow area at 55 ms for an untreated surface microchannel. Then, the shear force between vapor and liquid increases, and the liquid film breaks and enters the initial stage of dryout at 85 ms. With the passage of time, at 115 ms, the dryout area diffuses, and even enters the complete dry stage, which is shown as an unstable annular flow. In the dryout area, because the thin liquid film adheres to the wall tightly, the wall could not transfer heat to the mainstream liquid in time, and the incoming liquid could not be supplied, so the liquid between the wall and the vapor slug will be heated into superheated steam, which increases the heat transfer resistance between the wall and the mainstream, resulting in a sharp drop in the heat transfer coefficients and the outbreak of heat transfer crisis. At 145 ms, the new incoming liquid flushes the channel and starts a new flow period. In the annular flow stage, the dominant heat transfer mechanism of untreated surface microchannel is convective boiling.

It can be seen from Figure 7b that hydrophilic surface microchannel have less dryout spots, smaller wall dryout area, and improve heat transfer performance, compared with untreated surface microchannel. For super-hydrophilic microchannel, Figure 7c shows that there is no dryout phenomenon in the annular flow area, which demonstrates that the super-hydrophilic surface microchannel have excellent rewetting properties. When the liquid film is heated and thinned, there is enough liquid working fluid to fill the gap between the wall and the vapor slug, which effectively prevents the occurrence of dryout phenomenon, effectively solves the problem of heat transfer deterioration caused by local dryout patches, remarkably enhances heat transfer. Consequently, in the annular flow region, the effect of the liquid film is prominent, and the heat exchange mechanism of hydrophilic surface microchannel is no longer convective boiling but the thin liquid film evaporation.

### 3.4. Mechanism of Heat Transfer Enhancement in Super-Hydrophilic Microchannel

The behaviors of bubble nucleation, growth, and departure are closely related to the boiling heat transfer mechanism. So next we will conduct detailed analysis of bubble nucleation growth, departure behavior, and the forces on the vapor-liquid interface during the flow process.

#### 3.4.1. Bubble Nucleation Rate and the Number of Nucleation

Nucleate boiling refers to a heat transfer mode in which small bubbles are continuously formed and depart from the vaporized core on the surface of the microchannel. The bubble nucleation rate and the number of nucleation are the key of heat transfer enhancement.

The bubble nucleation process means that when two atoms form a gas molecule, they need to overcome the surface tension and pressure of the liquid to form a tiny gas volume unit. A good-wettability surface means that the solid surface tension is much greater than the liquid surface tension. Therefore, for a super-hydrophilic surface with superior wettability, it is more conducive for the solid surface atoms to overcome the surface tension of the liquid to form gas molecules, accelerate the nucleation growth rate, and thus promote the nucleation of bubbles.

In addition, nucleate boiling heat transfer is also closely related to the number of bubble nucleation. The shape and properties of the heating surface will directly affect the number of bubble nucleation. It can be seen from the SEM shown in Figure 2 of Section 2.2 above in this paper that the super-hydrophilic surface has more active nucleation cavities than the untreated surface, which can increase the number of bubble nucleation sites and enhance heat transfer.

#### 3.4.2. Bubble Departure Diameter and Departure Frequency

In the early stage of bubble growth, bubble growth rates can be characterized by bubbles departure diameter and departure frequency. Bubble departure diameter decreases with the decrease of the contact angle of the microchannel surface. Because the contact angle of the super-hydrophilic surface is very small, so the departure diameter is also very small, which is advantageous for nucleated bubbles to break the tension of the liquid surface tension with the help of solid surface tension and detach from the vaporization core site. This promotes two-phase flow process and strengthens heat transfer.

In addition to the bubble departure diameter that affects the heat transfer intensity, the bubble departure frequency is also an important parameter that determines the rate of heat transfer. The smaller the bubble departure diameter, the larger the departure frequency. For the super-hydrophilic surface, the bubble departure diameter is smaller, and the corresponding departure frequency is also faster, so the two-phase flow is enhanced, and the heat exchange is significantly improved.

#### 3.4.3. Force Analysis of the Vapor-Liquid Interface and Dryout Mechanism

**Figure 8:**
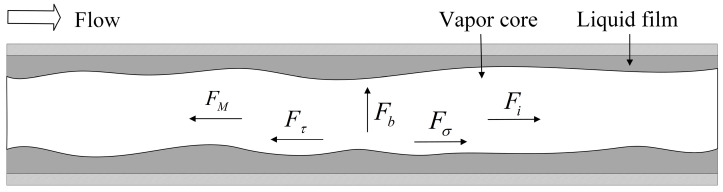
Schematic diagram of the force analysis of the vapor-liquid interface in the annular flow region in the microchannel.

Based on the transition of the two-phase flow patterns shown in Figure 5, Figure 6 and Figure 7, the force analysis of the vapor-liquid interface in the microchannel is carried out, and the mechanism of dryout is discussed. For the distribution and evolution of the vapor-fluid interface in the microchannel, it can be considered that it mainly depends on the interaction of the five forces, which are surface tension force $F_{\sigma }$, inertial force $F_{i}$, shear force $F_{\tau }$, evaporation momentum force $F_{M}$ and buoyancy force $F_{b}$. The schematic diagram of the force analysis of the vapor-liquid interface is shown in Figure 8.

The surface tension force $F_{\sigma }$ mainly depends on the contact angle and the size of the microchannel diameter. The smaller the contact angle and microchannel diameter, the greater the surface tension force. The inertial force $F_{i}$ is mainly determined by the mass flux. The greater the mass flux, the greater the inertial force. The direction of surface tension force and inertial force is the same as the flow direction, and these two forces are the driving force of two-phase flow. The shear force $F_{\tau }$ is related to the mass flux, the fluid viscosity and the microchannel diameter. In the case where the fluid viscosity and the microchannel diameter are unchanged, the greater the mass flux is, the greater the shear force. The evaporation momentum force $F_{M}$ mainly depends on the heat flux. The greater the heat flux, the greater the evaporation momentum force. The direction of the shear force and the evaporation momentum force is opposite to the direction of flow, and these two forces are the resistance of the two-phase flow. The buoyancy force $F_{b}$ is related to the gravity acceleration and can be ignored for two-phase flow in horizontal microchannels.

(a) The effect of high heat flux

The five forces interact and achieve dynamic equilibrium under normal flow conditions, stabilizing heat transfer. However, under higher heat flux conditions, for the untreated surface microchannel, the evaporation momentum force is greater and dominant. The sum of surface tension force and inertial force is less than the sum of evaporation momentum force and shear force, and the expression is as follows:

(6) $$F_{\sigma }+F_{i}<F_{M}+F_{\tau }$$

At this time, the balance of forces is broken, the flow is blocked, and the vapor-liquid interface is unevenly distributed, causing a lack of liquid and rupture of the liquid film, resulting in a dryout phenomenon.

For super-hydrophilic surface microchannel, the effect of surface tension is significant and greater than the increased evaporation momentum caused by high heat flux. Therefore, the sum of surface tension force and inertial force is greater than (or equal to) the sum of evaporation momentum force and shear force, which can be expressed as:

(7) $$F_{\sigma }+F_{i}\geq F_{M}+F_{\tau }$$

At this time, the flow is not blocked, the vapor-liquid interface maintains stable, and there is no dryout. This is consistent with the experiment conclusions in the previous Section 3.3.

(b) The effect of high mass flux

When the mass flux increases with other parameters remaining constant, both the inertial force and the shear force increase and the two forces offset each other. For an untreated surface microchannel, the forces are relatively balanced, but for a super-hydrophilic surface microchannel, the surface tension plays a leading role, so the sum of surface tension force and inertial force is greater than that of the evaporation momentum force and shear force. At this time, the flow of the two phases of vapor-liquid is enhanced under the action of composition of forces, and heat exchange is strengthened. Therefore, under high mass flux, the superiority of the heat exchange performance in the super-hydrophilic surface microchannel is more significant, which is consistent with the experiment conclusions described in Section 3.1.

## 4. Conclusions

This paper is based on experimental research on the flow boiling heat transfer characteristics and mechanism of three types of microchannels with a super-hydrophilic surface (θ ≈ 0°), a hydrophilic surface (θ = 43°) and an untreated surface (θ = 70°). The heat transfer performance advantages and mechanism of the super-hydrophilic surface microchannel are clarified, and the reasons for the dryout phenomenon are analyzed. The main conclusions obtained are as follows:

(1) The heat transfer performance of a super-hydrophilic surface microchannel is much better than that of an untreated one. At a lower mass flux of 740 kg/(m^2^s), the average heat transfer coefficients of the hydrophilic and super-hydrophilic microchannels are higher about 65% and 300% than those of the untreated one. At higher mass flux of 1265 kg/(m^2^s), the average heat transfer coefficients of the hydrophilic and the super-hydrophilic microchannels are about 85% and 325% higher than those of the untreated one.
(2) The mechanism of the heat transfer performance advantages of the super-hydrophilic surface microchannel is as follows: Compared with the untreated surface, the super-hydrophilic surface is rough and has more active nucleation cavities. As a result, the number of bubble nucleation sites generated in the super-hydrophilic surface microchannel is larger, and the bubble departure diameter is smaller, so the flow and heat transfer are enhanced. On the other hand, the contact angle of the super-hydrophilic surface microchannel is very small, so the departure diameter is small and the departure frequency is high, resulting in enhanced flow and heat transfer.
(3) Under high heat flux conditions, a dryout phenomenon emerges in the untreated surface microchannel, but there is no such appearance in a super-hydrophilic surface microchannel. This is because in the untreated surface microchannel, the evaporation momentum force increases and dominates at this time, causing the balance of the flow forces to be broken, the flow is blocked, the vapor-liquid interface is unevenly distributed, and dryout occurs. However, for a super-hydrophilic surface microchannel, due to its large surface tension, which can weaken the effect of the increase of evaporative momentum force, so that the sum of the surface tension force and inertial force is still greater than (or equal to) the sum of evaporation momentum force and shear force. At this time, the flow is not blocked, and the vapor-liquid interface remains uniform and stable to avoid dryout.

## Figures and Tables

**Table 1:** Dimension parameter of micro channel.

Parameter	Dimension
Number of channels	1
Hydraulic diameter, D_h_ [mm]	0.872
Channel length, L [mm]	78
Channel width, W_ch_ [mm]	1.6
Channel depth, H_ch_ [mm]	0.6

**Table 2:** Operating condition for single microchannel.

Experiment Parameters	Range	Unit
Surface contact angle	70, 43, 0	°
Saturation temperature	20	℃
Inter vapor quality	0–0.25	1
Mass flux	741–1267	kg/(m^2^s)
Heat flux	9–45	kW/m^2^

**Table 3:** Measurement error and uncertainty.

Parameter	Maximum Uncertainty (%)
Pressure, P [bar]	0.5
Differential pressure, ΔP [MPa]	0.5
Refrigerant temperature T_f_ [°C]	0.3
Wall temperature, T_w_ [°C]	0.5
Heat flux, q″ [kW/m^2^]	0.5
Mass velocity, G [kg/(m^2^s)]	5.7
Inlet vapor quality [–]	5.2
Local vapor quality [–]	7.2
Local heat transfer coefficient [kW/(m^2^K)]	12.5
Average heat transfer coefficient [kW/(m^2^K)]	12.5

“[–]” indicates that the parameter unit is 1.

## Data Availability

The data presented in this study are available on request from the corresponding author. The data are not publicly available due to privacy.

## Nomenclature

A_h_	heated area [m^2^]
c_p_	specific heat at constant pressure [kJ/(kg K)]
D_h_	hydraulic diameter [mm]
G	mass velocity [kg/(m^2^s)]
h	enthalpy [kJ/kg]
h_fg_	latent heat of vaporization [kJ/kg]
h_z_	local heat transfer coefficient [kW/(m^2^s)]
h	average heat transfer coefficient [kW/(m^2^s)]
H_tc_	distance between thermocouple and base of microchannel [m]
k	thermal conductivity [kW/(m K)]
L	length of microchannel [m]
p	pressure [bar]
$Q_{\mathrm{test}}$	power of heater [W]
$q^{\prime \prime }$	heat flux based on heated perimeter [kW/m^2^]
T	Temperature [°C]
x	vapor quality [-]
z	coordinate along microchannel [m]
**Greek symbols**	
β	channel aspect ratio [-]
Δ	difference [-]
**Subscripts**	
f	fluid
in	microchannel inlet
res	reservoir
sat	saturated
sub	subcooling
test	test section
w	wall
z	coordinate along microchannel

## References

[1] Mudawar I. (2011). Two-Phase Microchannel Heat Sinks: Theory, Applications, and Limitations. J. Electron. Packag..

[2] Valeh-e-Sheyda P., Rahimi M., Karimi E., Asadi M. (2013). Application of two-phase flow for cooling of hybrid microchannel PV cells: A comparative study. Energy Convers. Manag..

[3] Karayiannis T.G., Mahmoud M.M. (2017). Flow boiling in microchannels: Fundamentals and applications. Appl. Therm. Eng..

[4] Liang G., Mudawar I. (2020). Review of channel flow boiling enhancement by surface modification, and instability suppression schemes. Int. J. Heat Mass Transf..

[5] Yang F., Dai X., Peles Y., Cheng P., Khan J., Li C. (2014). Flow boiling phenomena in a single annular flow regime in microchannels (I): Characterization of flow boiling heat transfer. Int. J. Heat Mass Transf..

[6] Yang F., Dai X., Peles Y., Cheng P., Khan J., Li C. (2014). Flow boiling phenomena in a single annular flow regime in microchannels (II): Reduced pressure drop and enhanced critical heat flux. Int. J. Heat Mass Transf..

[7] Liu T.Y., Li P.L., Liu C.W., Gau C. (2011). Boiling flow characteristics in microchannels with very hydrophobic surface to super-hydrophilic surface. Int. J. Heat Mass Transf..

[8] Anwar Z., Palm B., Khodabandeh R. (2014). Flow boiling heat transfer and dryout characteristics of R152a in a vertical mini-channel. Exp. Therm. Fluid Sci..

[9] Lee S., Mudawar I. (2016). Transient characteristics of flow boiling in large micro-channel heat exchangers. Int. J. Heat Mass Transf..

[10] Lee S., Devahdhanush V.S., Mudawar I. (2018). Investigation of subcooled and saturated boiling heat transfer mechanisms, instabilities, and transient flow regime maps for large length-to-diameter ratio micro-channel heat sinks. Int. J. Heat Mass Transf..

[11] Morisaki M., Minami S., Miyazaki K., Yabuki T. (2021). Direct local heat flux measurement during water flow boiling in a rectangular minichannel using a MEMS heat flux sensor. Exp. Therm. Fluid Sci..

[12] Zhou K., Coyle C., Li J., Buongiorno J., Li W. (2017). Flow boiling in vertical narrow microchannels of different surface wettability characteristics. Int. J. Heat Mass Transf..

[13] Li W., Zhou K., Li J., Feng Z., Zhu H. (2018). Effects of heat flux, mass flux and two-phase inlet quality on flow boiling in a vertical superhydrophilic microchannel. Int. J. Heat Mass Transf..

[14] Li W., Chen Z., Li J., Sheng K., Zhu J. (2019). Subcooled flow boiling on hydrophilic and super-hydrophilic surfaces in microchannel under different orientations. Int. J. Heat Mass Transf..

[15] Bao Z.Y., Fletcher D.F., Haynes B.S. (2000). Flow boiling heat transfer of Freon R11 and HCFC123 in narrow passages. Int. J. Heat Mass Transf..

[16] Lin S., Kew P.A., Cornwell K. (2001). Flow Boiling of Refrigerant R141B in Small Tubes. Chem. Eng. Res. Des..

[17] Bertsch S.S., Groll E.A., Garimella S.V. (2009). Effects of heat flux, mass flux, vapor quality, and saturation temperature on flow boiling heat transfer in microchannels. Int. J. Multiph. Flow.

[18] Qu W., Mudawar I. (2003). Flow boiling heat transfer in two-phase micro-channel heat sinks––I. Experimental investigation and assessment of correlation methods. Int. J. Heat Mass Transf..

[19] Boye H., Staate Y., Schmidt J. (2007). Experimental investigation and modelling of heat transfer during convective boiling in a minichannel. Int. J. Heat Mass Transf..

[20] Mortada S., Zoughaib A., Arzano-Daurelle C., Clodic D. (2012). Boiling heat transfer and pressure drop of R-134a and R-1234yf in minichannels for low mass fluxes. Int. J. Refrig..

[21] Leão H.L.S.L., do Nascimento F.J., Ribatski G. (2014). Flow boiling heat transfer of R407C in a microchannels based heat spreader. Exp. Therm. Fluid Sci..

[22] Lee J., Mudawar I. (2005). Two-phase flow in high-heat-flux micro-channel heat sink for refrigeration cooling applications: Part II—heat transfer characteristics. Int. J. Heat Mass Transf..

[23] Huh C., Kim M.H. (2006). An experimental investigation of flow boiling in an asymmetrically heated rectangular microchannel. Exp. Therm. Fluid Sci..

[24] Lee P.-S., Garimella S.V. (2008). Saturated flow boiling heat transfer and pressure drop in silicon microchannel arrays. Int. J. Heat Mass Transf..

[25] Harirchian T., Garimella S.V. (2008). Microchannel size effects on local flow boiling heat transfer to a dielectric fluid. Int. J. Heat Mass Transf..

[26] Ducoulombier M., Colasson S., Bonjour J., Haberschill P. (2011). Carbon dioxide flow boiling in a single microchannel—Part II: Heat transfer. Exp. Therm. Fluid Sci..

[27] Lee S., Mudawar I. (2016). Investigation of flow boiling in large micro-channel heat exchangers in a refrigeration loop for space applications. Int. J. Heat Mass Transf..

[28] McNeil D.A., Raeisi A.H., Kew P.A., Hamed R.S. (2013). Flow boiling heat-transfer in micro to macro transition flows. Int. J. Heat Mass Transf..

[29] Balasubramanian K., Jagirdar M., Lee P.S., Teo C.J., Chou S.K. (2013). Experimental investigation of flow boiling heat transfer and instabilities in straight microchannels. Int. J. Heat Mass Transf..

[30] Ren C., Li W., Ma J., Huang G., Li C. (2021). Flow boiling in microchannels enhanced by parallel microgrooves fabricated on the bottom surfaces. Int. J. Heat Mass Transf..

[31] Yang Q., Shu B., Wang J., Guo Y. (2018). Experimental investigation on flow boiling heat transfer and flow patterns in a single micro-channel with large mass velocity. Exp. Therm. Fluid Sci..

[32] Nguyen T., Anh Ngo T., Duong Bang D., Wolff A. (2019). Optimising the supercritical angle fluorescence structures in polymer microfluidic biochips for highly sensitive pathogen detection: A case study on Escherichia coli. Lab Chip.

